# Gallic acid attenuates calcium calmodulin‐dependent kinase II‐induced apoptosis in spontaneously hypertensive rats

**DOI:** 10.1111/jcmm.13419

**Published:** 2017-12-20

**Authors:** Li Jin, Zhe Hao Piao, Chun Ping Liu, Simei Sun, Bin Liu, Gwi Ran Kim, Sin Young Choi, Yuhee Ryu, Hae Jin Kee, Myung Ho Jeong

**Affiliations:** ^1^ Heart Research Center of Chonnam National University Hospital Gwangju Korea; ^2^ Jilin Hospital Affiliated with Jilin University Chuanying Jilin China; ^3^ The Second Hospital of Jilin University Nanguan Changchun China

**Keywords:** gallic acid, spontaneously hypertensive rats (SHR), cardiac hypertrophy, Ca^2+^/calmodulin‐dependent protein kinase II, apoptosis

## Abstract

Hypertension causes cardiac hypertrophy and leads to heart failure. Apoptotic cells are common in hypertensive hearts. Ca^2+^/calmodulin‐dependent protein kinase II (CaMKII) is associated with apoptosis. We recently demonstrated that gallic acid reduces nitric oxide synthase inhibition‐induced hypertension. Gallic acid is a trihydroxybenzoic acid and has been shown to have beneficial effects, such as anti‐cancer, anti‐calcification and anti‐oxidant activity. The purpose of this study was to determine whether gallic acid regulates cardiac hypertrophy and apoptosis in essential hypertension. Gallic acid significantly lowered systolic and diastolic blood pressure in spontaneously hypertensive rats (SHRs). Wheat germ agglutinin (WGA) and H&E staining revealed that gallic acid reduced cardiac enlargement in SHRs. Gallic acid treatment decreased cardiac hypertrophy marker genes, including atrial natriuretic peptide (*ANP*) and brain natriuretic peptide (*BNP*), in SHRs. The four isoforms, α, β, δ and γ, of *CaMKII* were increased in SHRs and were significantly reduced by gallic acid administration. Gallic acid reduced cleaved caspase‐3 protein as well as *bax*,* p53* and *p300* mRNA levels in SHRs. *CaMKII* δ overexpression induced *bax* and *p53* expression, which was attenuated by gallic acid treatment in H9c2 cells. Gallic acid treatment reduced DNA fragmentation and the TUNEL positive cells induced by angiotensin II. Taken together, gallic acid could be a novel therapeutic for the treatment of hypertension through suppression of CaMKII δ‐induced apoptosis.

## Introduction

Hypertension is a major cardiovascular risk factor that leads to atherosclerosis, cardiac hypertrophy, heart failure and stroke. Spontaneously hypertensive rats (SHRs) are a well‐established genetic animal model of hypertension that mimics essential hypertension in humans 1. Cardiac hypertrophy is typically present in hypertensive rats 2. Hypertension induces left ventricular hypertrophy (LVH), which is characterized by increased cardiomyocyte size, increased protein synthesis, activation of foetal gene programmes and reorganization of sarcomere structure 3. Hypertension is associated with myocardial apoptosis, which is a process of programmed cell death. Increased apoptosis occurs in the heart tissue of SHRs 4, 5. It was recently shown that endoplasmic reticulum (ER) stress induces apoptosis in SHRs 6. ER stress activates Ca^2+^/calmodulin‐dependent protein kinase II (CaMKII) *via* several pathways 7.

CaMKII is involved in the development of pathological cardiac hypertrophy and heart failure 8, 9. CaMKII is currently recognized as a key mediator of cardiovascular disease. CaMKII δ and γ isoforms are expressed in the heart 10, whereas CaMKII α and β isoforms are expressed in the brain. We recently reported that *CaMKII* α mRNA and protein expression are induced in angiotensin II‐treated vascular smooth muscle cells 11. This implicates CaMKII α as having a role in hypertension. CaMKII δ has two forms, CaMKII δ_B_ and CaMKII δ_C_. Mice that overexpressed nuclear CaMKII δ_B_ were shown to develop cardiac hypertrophy and dilated cardiomyopathy, whereas transgenic mice overexpressing cytoplasmic CaMKII δ_C_ exhibited dilated cardiomyopathy and heart failure 12. Double‐knockout mice deficient in CaMKII δ and γ exhibited adverse cardiac remodelling 13. CaMKII can lead to apoptosis 9. For example, CaMKII δ_C_ transgenic mice develop heart failure with cardiomyocyte apoptosis. Additionally, there is evidence that inhibition of CaMKII prevents cardiac hypertrophy 14 and hypertension 15.

Gallic acid has been reported to have anti‐calcification 16, anti‐hypertension 17, anti‐hypertrophy 18, anti‐obesity 19 and anti‐oxidant activity 20. However, the effect of gallic acid on apoptosis in hypertension has not been determined.

In the present study, we showed that gallic acid reduces high blood pressure and apoptosis in SHRs. We report that gallic acid down‐regulates *CaMKII* expression and apoptosis‐related genes in hypertensive hearts, suggesting that it has potential as a novel therapeutic for hypertension.

## Materials and methods

### Animal treatment and blood pressure measurements

All animal procedures were approved by the Animal Experimental Committee of the Chonnam National University Medical School (CNU IACUC‐H‐2014‐48). Wistar–Kyoto rats (WKY, 4‐week‐old males, *n* = 14) and spontaneously hypertensive rats (SHRs, 4‐week‐old males, *n* = 28) were obtained from SLC Company (Shizuoka, Japan). To investigate the effect of gallic acid, rats were divided into three groups: WKYs, SHRs and SHRs plus gallic acid. Gallic acid (1% in tap water) was administered to SHRs for 4 months.

Blood pressures were measured as previously described 21. Briefly, systolic and diastolic blood pressures of wakeful rats were measured using the tail‐cuff method (Visitech Systems, Apex, North Carolina, USA, BP‐2000).

### Left ventricular hypertrophy

After killing, the hearts from the rats were obtained, the atrium was removed and the left ventricle was isolated. Left ventricular hypertrophy was expressed as a ratio of the left ventricular weight to tibia length (mg/mm).

### Wheat germ agglutinin staining

Heart tissues were fixed in 4% paraformaldehyde at room temperature, embedded in paraffin and cut into 3‐μm thin sections. To determine the cross‐sectional area of the myocardium, wheat germ agglutinin (WGA) staining was used as previously described 22. Antigen retrieval in deparaffinized heart slides was performed with citrate buffer. Endogenous peroxidase activity was eliminated by application of 3% hydrogen peroxide (H_2_O_2_). After blocking with 1% bovine serum albumin (BSA), tissue sections were incubated with wheat germ agglutinin Alexa Fluor 488 (1:200) for 1 hr. After washing three times (PBS), the slides were mounted with a mounting medium. Stained cells were visualized using a fluorescence microscope.

### Haematoxylin and eosin (H&E) staining

Heart slides were deparaffinized three times using xylene and hydrated through a series of decreasing ethanol concentrations (100%, 95%, 90%, 80% and 70%). Tissues were incubated in Gill's haematoxylin V for 5 min. and washed with tap water for 5 min. After dipping in 95% ethanol for 2 min., the tissues were incubated in Eosin Y for 1 min. Next, the tissues were gradually dehydrated using 95% ethanol, 100% ethanol, and xylene and finally mounted with Canada balsam. Photomicrographs were obtained using Eclipse Ti‐U microscope (Nikon, Miyagi, Japan).

### Terminal deoxynucleotidyl transferase dUTP nick end labelling (TUNEL) staining

The TUNEL assay was performed according to the manufacturer's protocol (Promega, California, USA). H9c2 cells were seeded on a coverslip and serum starved overnight. Cells were treated with vehicle or gallic acid (50 μM) under angiotensin II stimulus (100 μM). Cells were fixed with 4% paraformaldehyde at 4°C and permeabilized using 0.2% Triton X‐100 in PBS. After equilibration, cells were labelled using TdT reaction mix for 60 min. at 37°C. To visualize the nuclei, cells were stained with DAPI. Apoptotic cells were analysed using a fluorescence microscope.

### DNA fragmentation

H9c2 cells were seeded into 6‐cm dishes (8 × 10^5^ per well). H9c2 cells were serum starved for 12 hrs and incubated with the angiotensin II stimulus (100 μM) in the presence/absence of gallic acid (25 μM) for 24 hrs. Cells were harvested using a 1× dissociation reagent (TrypLE Expression, Gibco, NY, USA) and washed with PBS. Cell pellets were lysed in 250 μl of DNA extraction buffer (10 mM NaCl, 20 mM EDTA, 50 mM Tris‐HCl, pH 8.0, 1% SDS and 20 μg/ml RNase A) for 2 hrs at 37°C. The cells were treated with 100 μg/ml of proteinase K for 1 hr at 65°C. To isolate DNA, 250 μl of phenol:chloroform:isoamyl alcohol (25:24:1) was added to the lysates. After centrifugation, a final concentration of 200 mM NaCl and two volumes of ice‐cold 100% ethanol were added to the supernatant at −20°C for 1 hr to precipitate the DNA. The DNA pellet was dissolved in 50 μl of 1 × TE buffer (10 mM Tris‐HCl, 1 mM EDTA, pH 8.0). DNA was subjected to electrophoresis on a 1.7% agarose gel with ethidium bromide.

### Western blot analysis

Western blots were performed as previously described 23. Protein lysates from left ventricular or kidney cortex tissues were prepared with RIPA buffer (150 mM NaCl, 1% Triton X‐100, 1% sodium deoxycholate, 50 mM Tris‐HCl, pH 7.5, 2 mM EDTA, 1 mM PMSF, 1 mM DTT, 1 mM Na_3_VO_4_, 5 mM NaF) containing protease inhibitors. Proteins were separated by 10% SDS‐PAGE and subsequently transferred to polyvinylidene difluoride (PVDF) membranes. The membranes were exposed to the indicated antibodies and developed using Immobilon Western Detection Reagents (Millipore, Billerica, MA, USA). Bio‐ID software was used to quantify protein expression (Vilber Lourmat, Eberhardzell, Germany).

Antibodies for Bax (sc‐526) and GAPDH (sc‐32233) were purchased from Santa Cruz Biotechnology (Dallas, TX, USA). Antibodies for caspase‐3 (9662) and pan‐CaMKII (44365) were purchased from Cell Signaling Technology (Danvers, MA, USA). CaMKII δ (GTX111401) antibody was purchased from Genetex (Irvine, CA, USA). CaMKII γ (ab88670) antibody was purchased from Abcam (Cambridge, UK).

### Transfection

H9c2 cells were transfected with pcDNA3‐CaMKIIδ full constructs using Plus and Lipofectamine reagents according to the manufacturer's protocol (Invitrogen, Massachusetts, USA). pcDNA3‐CaMKIIδ constructs were kindly provided by Prof. Eric N. Olson (UT Southwestern Medical Center, Dallas, Texas, USA).

### RNA isolation and real‐time PCR

The isolation and determination of mRNA levels from all animals (*n* = 14 per group) were performed as follows. Total RNA was isolated using TRIzol reagent (Invitrogen Life Technologies). From each isolation, 1 μg of RNA was used in a reverse transcription reaction with TOPscript RT DryMIX (Enzynomics, Daejeon, South Korea). mRNA levels were quantified using a SYBR Green PCR kit (Enzynomics). Gene expression levels were compared with that of *18S* using the 2^−ΔΔct^ method. The sequences of the PCR primers were as follows: *18S* rRNA, sense, 5′‐CATTCGAACGTCTGCCCTAT‐3′ and antisense, 5′‐ GCCTTCCTTGGATGTGGTAG‐3′; *GAPDH*, sense, 5′‐AACCCATCACCATCTTCCAGGAGC‐3′ and antisense, 5′‐ ATGGACTGTGGTCATGAGCCCTTC‐3′; *ANP*, sense, 5′‐GCTCGAGCAGATCGCAAAAG‐3′ and antisense, 5′‐GAGTGGGAGAGGTAAGGCCT‐3′; *BNP*, sense, 5′‐GACGGGCTGAGGTTGTTTTA‐3′ and antisense, 5′‐ACTGTGGCAAGTTTGTGCTG‐3′; *CaMKII*α, sense, 5′‐ACAGAGCAGCTGATCGAAGC‐3′ and antisense, 5′‐AGGTGGATGTGAGGGTTCAG‐3′; *CaMKII*β, sense, 5′‐GGGACACCGTTACTCCTGAA‐3′ and antisense, 5′‐ CTCCCTTGAGCTTCCTCCTT‐3′; *CaMKII* δ, sense, 5′‐ACAACCCTGACGGAAACAAG‐3′ and antisense, 5′‐CCACTAAGTTGCCCAATGCT‐3′’; *CaMKII* γ, sense, 5′‐ ACAACCCTGACGGAAACAAG‐3′ and antisense, 5′‐CCACTAAGTTGCCCAATGCT‐3′; *bax*, sense, 5′‐GAGAGGATGGCTGGGGAGACAC‐3′ and antisense, 5′‐ GAGGAAGTCCAGTGTCCAGCCC‐3′; *p53*, sense, 5′‐CACAGTCGGATATGAGCATCGAGC‐3′ and antisense, 5′‐ CACAACTGCACAGGGCATGTCTTC‐3′; *p300*, sense, 5′‐AGCCAAAGAAAAAGATTTTCA‐3′ and antisense, 5′‐ ACATCACTGGGTCAATTTCTT‐3′. The mRNA levels were normalized to *18S* rRNA or *GAPDH*.

### Statistical analysis

For statistical analysis, Student's *t*‐test was performed using GraphPad Prism version 5.0. Data are presented as means ± S.D. *P* values below 0.05 were considered statistically significant.

## Results

### Gallic acid lowers blood pressure in spontaneously hypertensive rats

**Figure 1:**
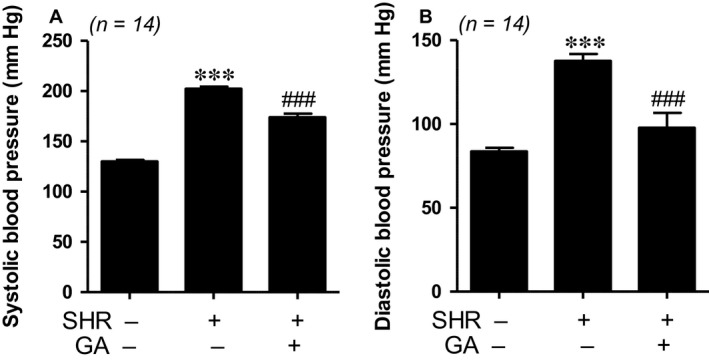
Gallic acid lowers blood pressure in spontaneously hypertensive rats. (**A, B**) Systolic and diastolic blood pressures were measured in three groups: WKY rats, SHRs and SHRs plus gallic acid. ***P <* *0.001 *versus* WKY rats. ^###^ *P *<* *0.001 *versus* SHRs.

We recently reported that gallic acid lowered blood pressure in N^G^‐nitro‐L‐arginine methyl ester (_L_‐NAME)‐induced hypertension 17. Spontaneously hypertensive rats (SHRs) are a representative animal model of essential hypertension. Thus, we sought to determine whether gallic acid affects hypertension in SHRs. SHRs presented significantly elevated systolic and diastolic blood pressures in comparison with those in WKY rats (202.4 ± 6.6 mm Hg *versus* 130.1 ± 5.7 mm Hg and 137.7 ± 24.0 mm Hg *versus* 83.6 ± 12.0 mm Hg, respectively). Long‐term treatment with gallic acid (4 months) significantly reduced systolic and diastolic blood pressure (Fig. 1A and B).

### Gallic acid attenuates left ventricular hypertrophy in spontaneously hypertensive rats

**Figure 2:**
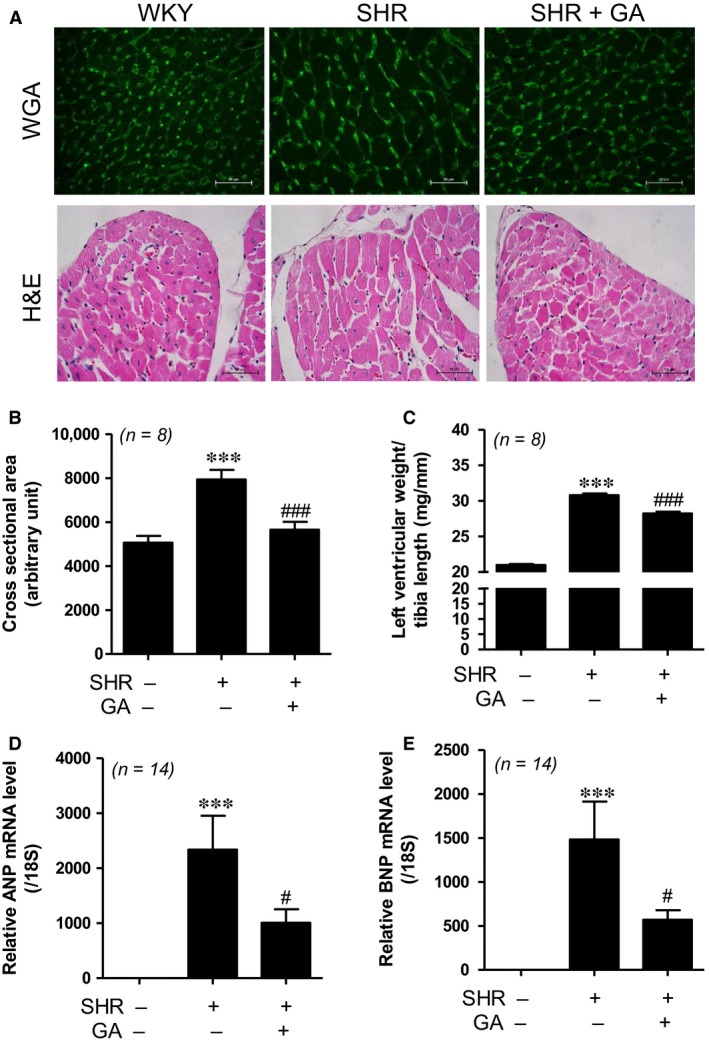
Gallic acid attenuates left ventricular hypertrophy in spontaneously hypertensive rats (**A**) WGA (top panel) and H&E (bottom panel) staining performed to evaluate the increased size in myocytes. Scale bar = 50 μm. (**B**) Cross‐sectional area of the left ventricle was evaluated (*n *=* *8 per group). ***P <* *0.001 compared with WKY rats. ^###^ *P *<* *0.001 *versus* SHRs. (**C**) The ratio of the left ventricular weight to tibia length (mg/mm) in WKY, SHR, SHR+GA (*n *=* *8 per group). ***P <* *0.001 compared with WKY rats. ^###^ *P *<* *0.001 *versus* SHRs. (**D, E**) The mRNA levels of *ANP* and *BNP* were evaluated by real‐time RT‐PCR from three groups (*n *=* *14). The transcript levels were normalized to those for *18S* and presented as relative values. ***P <* *0.001 *versus* WKY rats. ^#^ *P *<* *0.05 *versus* SHRs.

To identify whether gallic acid could reduce left ventricular hypertrophy, wheat germ agglutinin (WGA) and H&E staining were performed to measure cardiomyocyte area. As shown in Figure 2A, both staining techniques determined that SHRs had enlarged cardiomyocytes compared to WKY rats. The increased size was reduced by gallic acid administration (Fig. 2B).

The ratio of the left ventricular weight to tibia length was significantly increased in SHRs compared to WKY control. Gallic acid treatment attenuated the ratio of left ventricular weight to tibia length in SHRs (Fig. 2C).

To investigate whether expression profiles of cardiac hypertrophy markers are abnormal in SHRs, we performed real‐time RT‐PCR. Atrial natriuretic peptide (*ANP*) and brain natriuretic peptide (*BNP*) mRNA levels were significantly augmented in SHRs compared to those in WKY rats. This increase was decreased by gallic acid treatment (Fig. 2D and E).

### Gallic acid down‐regulates expression of Ca^2+^/calmodulin‐dependent protein kinase II in spontaneously hypertensive rats

**Figure 3:**
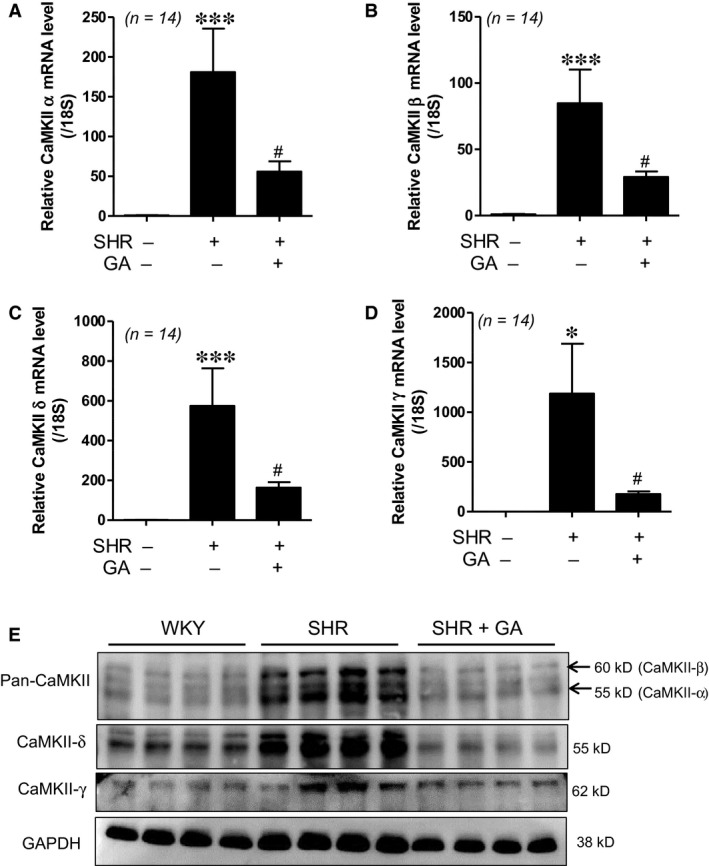
Gallic acid down‐regulates expression of Ca^2+^/calmodulin‐dependent protein kinase II in spontaneously hypertensive rats. (**A–D**) Real‐time RT‐PCR was performed in LV tissues from WKY rats, SHRs and SHRs plus gallic acid. The mRNA levels of *CaMKII* α, β, δ and γ were determined. The transcript levels were normalized to those for *18S*. ***P <* *0.001 *versus* WKY rats. ^#^ *P *<* *0.05 *versus* SHRs. (**E**) Representative immunoblots. Western blot analysis for pan‐CaMKII, CaMKII δ and CaMKII γ in LV tissues from WKY rats, SHRs and SHRs plus gallic acid (*n *=* *8 per group). Antibodies for pan‐CaMKII detected CaMKII β (60 kD) and CaMKII α (55 kD).

Ca^2+^/calmodulin‐dependent protein kinase II (CaMKII) is associated with pathological cardiac hypertrophy 9, 24. CaMKII has four isoforms (α, β, δ and γ). We evaluated the mRNA levels of *CaMKII* α, β, δ and γ in left ventricular (LV) tissues. mRNA levels for all four isoforms of *CaMKII* were increased in SHRs compared to those in WKY rats. The increase was significantly reduced by gallic acid treatment (Fig. 3A–D). In addition, we performed Western blotting using antibodies of the four isoforms. Pan‐CaMKII antibodies detected CaMKII β and CaMKII α forms. As shown in Figure 3E, the protein expression of CaMKII α, β, δ and γ was increased in SHR hearts when compared to WKY hearts. Gallic acid treatment decreased protein levels of CaMKII α, β, δ and γ.

### Gallic acid reduces apoptosis in spontaneously hypertensive rats

**Figure 4:**
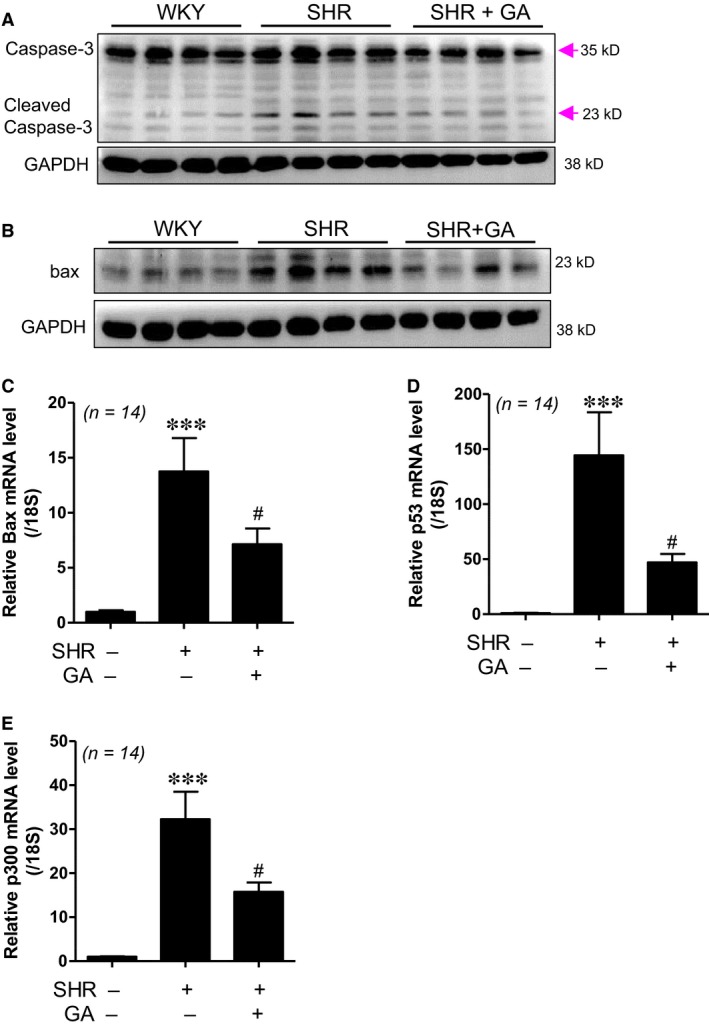
Gallic acid reduces apoptosis in spontaneously hypertensive rats. (**A**) Representative immunoblots. Western blot analysis for the expression of cleaved caspase‐3 performed in LV tissues from WKY rats, SHRs and SHRs plus gallic acid (*n *=* *8 per group). Caspase‐3 and cleaved caspase‐3 have sizes of 35 kD and 23 kD, respectively. GAPDH was used as a loading control. (**B**) Western blot analysis for the expression of bax protein was performed in LV tissues from WKY rats, SHRs and SHRs plus gallic acid. (**C–E**) The mRNA levels of *bax*,* p53* and *p300* were evaluated by real‐time RT‐PCR. The transcript levels were normalized to those for *18S*. ***P <* *0.001 *versus* WKY rats. ^#^ *P *<* *0.05 *versus* SHRs.

Apoptosis is involved in the development of hypertension 4. To determine whether gallic acid could affect apoptosis, we performed Western blot analysis. Cleaved caspase‐3 protein expression was higher in SHRs than in WKY rats. This increase was significantly reduced by gallic acid treatment (Fig. 4A and Fig. S1A). We observed that gallic acid treatment decreased bax protein expression in SHRs compared to that in WKY rats (Fig. 4B and Fig. S1B). In addition, *bax* mRNA levels in SHRs were effectively decreased by gallic acid administration (Fig. 4C). We further investigated apoptosis‐related gene expression. Transcript levels of *p53* and *p300* were enhanced in SHRs compared to those in WKY rats. The increase was significantly reduced by gallic acid treatment (Fig. 4D and E).

### Gallic acid suppresses apoptosis induced by CaMKII δ overexpression or angiotensin II stimulus in H9c2 cells

Cardiomyocyte apoptosis is associated with CaMKII in heart cells 9, 25. We examined the protein expression of the four isoforms of CaMKII in angiotensin II‐treated H9c2 cells. As shown in the Figure S2A–E, CaMKII δ protein levels were increased in response to angiotensin II. Therefore, we decided to focus on the role of CaMKII δ on apoptosis.

**Figure 5:**
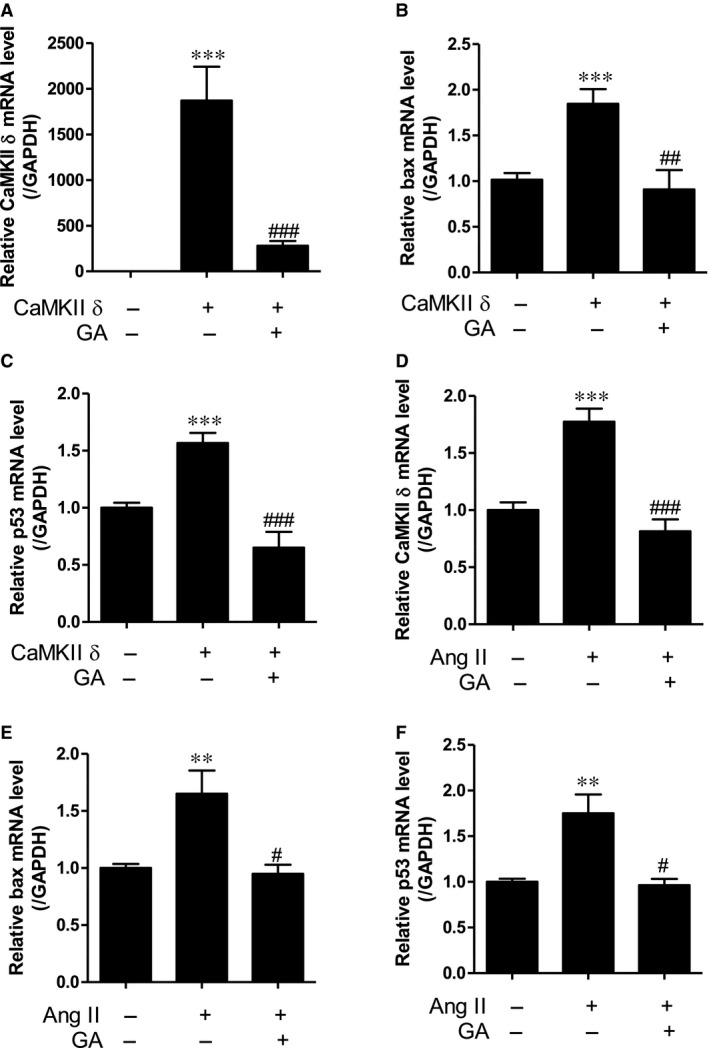
Gallic acid suppresses apoptosis induced by *CaMKII* δ overexpression or angiotensin II stimulus in H9c2 cells. (**A–C**) H9c2 cells were transfected with empty vector or pcDNA3‐CaMKIIδ and then were treated with gallic acid for 6 hrs. The expression of *CaMKII* δ (**A**), *bax* (**B**) and *p53* (**C**) was assayed by real‐time RT‐PCR. The transcript levels were normalized to those for *GAPDH*. ***P <* *0.001 *versus* empty vector. ^##^ *P *<* *0.01 and ^###^ *P *<* *0.001 *versus* CaMKII δ transfection. (**D–F**) H9c2 cells were incubated with angiotensin II (100 μM) and then treated with gallic acid (100 μM) for 6–9 hrs. *CaMKII* δ (**D**), *bax* (**E**) and *p53* (**F**) transcript levels were determined by real‐time RT‐PCR. **P <* *0.01 and ***P <* *0.001 *versus* empty vector. ^#^ *P *<* *0.05 and ^###^ *P *<* *0.001 *versus* angiotensin II‐treated group. Data represent the means ± standard error (S.E.) for at least four independent experiments.

To determine whether CaMKII δ could affect apoptosis, we performed real‐time RT‐PCR. Transfection with *CaMKII* δ dose‐dependently increased *CaMKII* δ mRNA levels (Fig. S3A). *CaMKII* δ overexpression increased *bax* and *p53* transcript levels (Fig. S3B and C). We next investigated the effect of gallic acid on CaMKII‐mediated apoptosis. Gallic acid treatment significantly reduced the up‐regulated *CaMKII* δ mRNA levels (Fig. 5A). In addition, it decreased *bax* and *p53* transcript levels, which were up‐regulated by CaMKII δ overexpression (Fig. 5B and C). We examined the expression of apoptosis‐related genes after angiotensin II application. We observed that angiotensin II induced *CaMKII* δ, *bax* and *p53* mRNA levels in a dose‐dependent manner (Fig. S4A–C). To further investigate the effect of gallic acid on hypertension‐induced apoptosis, H9c2 cells were exposed to angiotensin II and then treated with gallic acid. Gallic acid treatment significantly suppressed the increase in *CaMKII* δ, *p53* and *bax* mRNA levels induced by angiotensin II (Fig. 5D–F).

### Gallic acid reduces angiotensin II‐induced apoptosis as determined by TUNEL assay and DNA fragmentation

**Figure 6:**
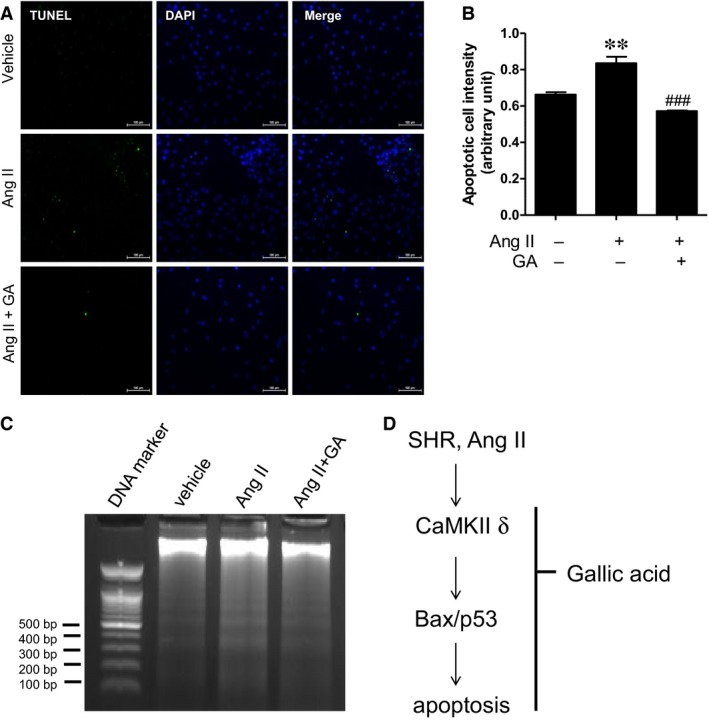
Gallic acid reduces angiotensin II‐induced apoptosis as determined by TUNEL assay and DNA fragmentation. (**A**) Representative images of TUNEL staining. H9c2 cells were serum starved for 24 hrs and were treated with gallic acid (50 μM) in the presence or absence of angiotensin II (10 μM) for 36 hrs. Green and blue colours indicate apoptotic cells and nuclei in H9c2 cells. (**B**) Quantification of positive TUNEL staining. **P <* *0.01 *versus* vehicle group. ^###^ *P *<* *0.001 *versus* the angiotensin II‐treated group. Data represent the means ± S.E. of at least three independent experiments. (**C**) Representative DNA fragmentation of angiotensin II (100 μM)‐treated H9c2 cells in the presence or absence of gallic acid (25 μM). H9c2 cells lysates were treated with RNase A and proteinase K before DNA extraction. Apoptosis was determined by 1.7% agarose gel electrophoresis; 100 bp DNA ladder was used as a loading control.

To investigate the effect of gallic acid on angiotensin II‐induced apoptosis, the TUNEL assay was performed on H9c2 cells. TUNEL positive cells were more numerous in angiotensin II‐treated groups, and this increase was reduced by gallic acid treatment (Fig. 6A and B). In addition, we confirmed the antiapoptotic effect of gallic acid in H9c2 cells using DNA fragmentation. Angiotensin II (100 μM) stimulus increased DNA fragmentation in H9c2 cells, which was reduced by 25 μM gallic acid treatment (Fig. 6C).

## Discussion

We have clearly demonstrated that gallic acid attenuates cardiac hypertrophy and apoptosis in essential hypertension. Our results showed that long‐term treatment with gallic acid reduced high blood pressure in spontaneously hypertensive rats (SHRs). This finding was consistent with our recent study, in which gallic acid lowered high blood pressure in a nitric oxide synthase inhibition‐induced hypertension mouse model 17. Hypertension usually accompanies left ventricular hypertrophy (LVH) 26. Gallic acid decreased the enhanced cardiomyocyte size in SHRs, as determined by WGA staining. This result is in agreement with our previous data and that of another group 18, 27. Gallic acid has been shown to prevent isoproterenol‐induced cardiac hypertrophy. Gallic acid was also reported to reduce left ventricular hypertrophy (LVH) in streptozotocin‐induced diabetes. Based on the results of the present study, we suggest that gallic acid can regulate cardiac hypertrophy caused by heart pathologies such as hypertension.

Calcium signalling is an important regulator of contraction in cardiovascular diseases, including cardiac hypertrophy, hypertension and heart failure 28. Ca^2+^/calmodulin‐dependent protein kinase II (CaMKII) is a multifunctional kinase involved in maladaptive cardiac remodelling 9. CaMKII inhibition prevented angiotensin II‐mediated arterial hypertension 29. This led us to suggest that CaMKII may be a key mediator of hypertension. We observed that levels of four isoforms (α, β, δ and γ) of *CaMKII* were increased in SHRs compared to those in WKY rats. In accordance with our results, *CaMKII* δ*3* isoform expression was found to be higher in hearts of dilated cardiomyopathy patients 30. Hagemann and colleagues reported that SHRs exhibited increased mRNA levels of *CaMKII* δ*4* (δ_*D*_) and *CaMKII* δ*9* (δ_*I*_), in embryonic and adult cardiac tissues, respectively 31. CaMKII δ2 (δ_C_) was found to be involved in the pathogenesis of dilated cardiomyopathy and heart failure 12. Similarly, cardiac‐specific *CaMKII* δ*3* (δ_*B*_) transgenic mice presented cardiac hypertrophy and dilation with decreased ventricular function 32.

The cardiac apoptotic pathway is linked to the development of hypertension 33. Apoptosis was increased in the heart of SHRs 4. Bax, p53, angiotensin II and ischaemia are known as proapoptotic factors in arterial hypertension 33. As shown in Figure 6C, hypertensive stimulus induced expression of apoptosis‐related genes, including *CaMKII* δ, *bax* and *p53,* and this increase was inhibited by gallic acid treatment. We also found that *CaMKII* δ overexpression induced *bax* and *p53* mRNA expression in angiotensin II‐treated H9c2 cells. In agreement with our results, the expression of constitutively active *CaMKII* δ*c* promoted cardiomyocyte apoptosis 34. In addition, KN‐93, a CaMKII inhibitor, attenuated *p53* and *bax* expression in a dilated cardiomyopathy model 35.

In this study, we clearly demonstrated that gallic acid reduces *CaMKII* α, β, δ and γ mRNA levels in the hearts of SHRs. We investigated the mitochondrial‐dependent pathway, including *bax* and activated caspase‐3 in SHRs. Gallic acid decreased *bax* mRNA and protein expression as well as cleaved caspase‐3 protein in SHRs. Furthermore, the TUNEL assay and DNA fragmentation demonstrated that gallic acid reduces angiotensin II‐induced apoptosis in H9c2 cells.

Furthermore, gallic acid resulted in the suppression of *p53* and *p300* mRNA levels in SHRs. Histone acetyltransferase p300 regulates p53‐dependent apoptosis after DNA damage 36. Overexpression of p300 efficiently induces acetylation of p53. In the present study, our findings suggest that gallic acid could prevent cardiac apoptosis. However, gallic acid acts as an anti‐cancer agent through induction of apoptosis 37, 38. This suggests that gallic acid could also be used to treat cancer.

Gallic acid is a trihydroxybenzoic acid and is a type of phenolic acid. Angiotensin‐converting enzyme (ACE) affects the development of hypertension. One of the most widely used drug types for treatment of hypertension is ACE inhibitors. Sharifi N. *et al*. reported that medicinal plants with ACE inhibition activity could be used to treat hypertension based on the results of an *in vitro* assay 39. Another study has shown that high phenol content extracts from *Thymus serpyllum L*. reduced systolic and diastolic blood pressure in SHRs 40. The possible mechanism for gallic acid reducing blood pressure could be the inhibition of angiotensin II type I receptor (Jin, Li. *et al*. in review). A second potential mechanism is the suppression of renin activity. In fact, gallic acid and epicatechin gallate have been found to exhibit renin‐inhibitory activity 41.

In conclusion, we have demonstrated that gallic acid treatment attenuates cardiac hypertrophy and apoptosis *via* down‐regulation of *CaMKII* δ and apoptosis‐related genes in an essential hypertension rat model. We suggest that gallic acid can be considered as a novel therapeutic for hypertension.

## Conflicts of interest

The authors declare no conflict of interest.

## Supporting information


**Fig S1.** Gallic acid reduces apoptosis in spontaneously hypertensive rats.
**Fig. S2.** *CaMKII δ* protein levels are increased in angiotensin II‐treated H9c2 cells.
**Fig. S3.** The forced expression of *CaMKII δ* increases *CaMKII δ*,* bax*, and *p53* mRNA levels in H9c2 cells.
**Fig. S4.** Angiotensin II stimulus increases mRNA levels of *CaMKII δ*,* bax*, and *p53* in H9c2 cells.
