# Supplementary material for: Gallic acid attenuates calcium calmodulin‐dependent kinase II‐induced apoptosis in spontaneously hypertensive rats
Source: J Cell Mol Med. 2017 Dec 20;22(3):1517–26. doi: 10.1111/jcmm.13419 (PMC5824377; doi:10.1111/jcmm.13419)

Gallic Acid Attenuates Calcium Calmodulin-Dependent Kinase II-Induced Apoptosis in Spontaneously Hypertensive Rats

Li Jin[1,2+], Zhe Hao Piao[3+], Chun Ping Liu[2], Simei Sun[1], Bin Liu[3], Gwi Ran Kim[1], Sin Young Choi[1], Yuhee Ryu[1], Hae Jin Kee[1]*, and Myung Ho Jeong[1]*

Supplementary Figure legends

Figure 1. Gallic acid reduces apoptosis in spontaneously hypertensive rats.

(A) Cleaved caspase 3 protein expression quantified by densitometry (n=8 per group). **(B)** Bax protein quantified by densitometry (n=8 per group). *P<0.05 and ***P<0.001 versus WKY rats. #P<0.05 and ##P<0.01 versus SHRs.

Figure 2. *CaMKII δ* protein levels are increased in angiotensin II-treated H9c2 cells.

(A) Representative western blot. H9c2 cells were treated with angiotensin II (100 µM, Ang II) for 12 h. Pan-CaMKII, CaMKII δ, and CaMKII γ antibodies were used. Pan-CaMKII antibody detected two isoforms (CaMKII β and CaMKII α). **(B–E)** The four isoforms of CaMKII protein levels quantified by densitometry. *P<0.05 and ***P<0.001 versus WKY rats. #P<0.05 and ##P<0.01 versus SHRs.

Figure 3. The forced expression of *CaMKII δ* increases *CaMKII δ*, *bax*, and *p53* mRNA levels in H9c2 cells.

(A–C) H9c2 cells were transfected with empty vector or pcDNA3-CaMKIIδ and total RNA was extracted. The transcript levels of *CaMKII δ*, *bax*, and *p53* were determined by real-time RT-PCR.

*P<0.05, *P<0.01, and ***P<0.001 versus vehicle-treated group. Data represent the means ± SE of at least 3 independent experiments.

Figure 4. Angiotensin II stimulus increases mRNA levels of *CaMKII δ*, *bax*, and *p53* in H9c2 cells.

(A–C) H9c2 cells were treated with the indicated concentration of angiotensin II (100 μM) for 36 h. The transcript levels of *CaMKII δ*, *bax*, and *p53* were determined by real-time RT-PCR. *P<0.05 versus vehicle-treated group. Data represent the means ± SE of at least 3 independent experiments.

Supplementary Figure 1

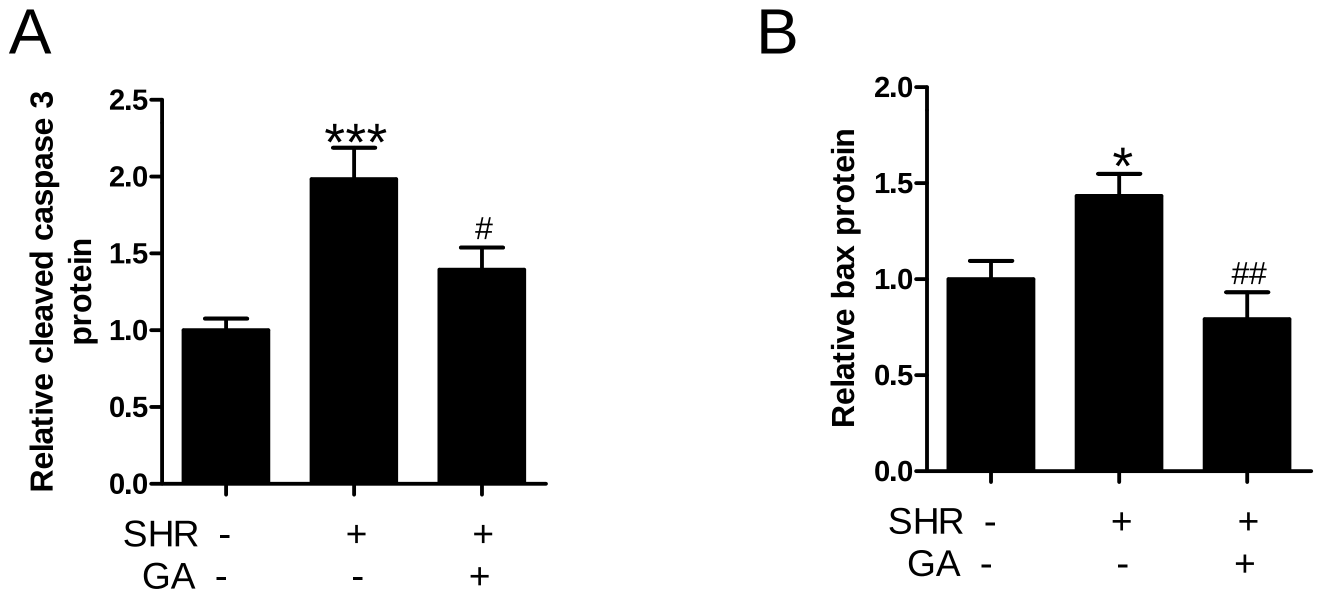

Supplementary Figure 2

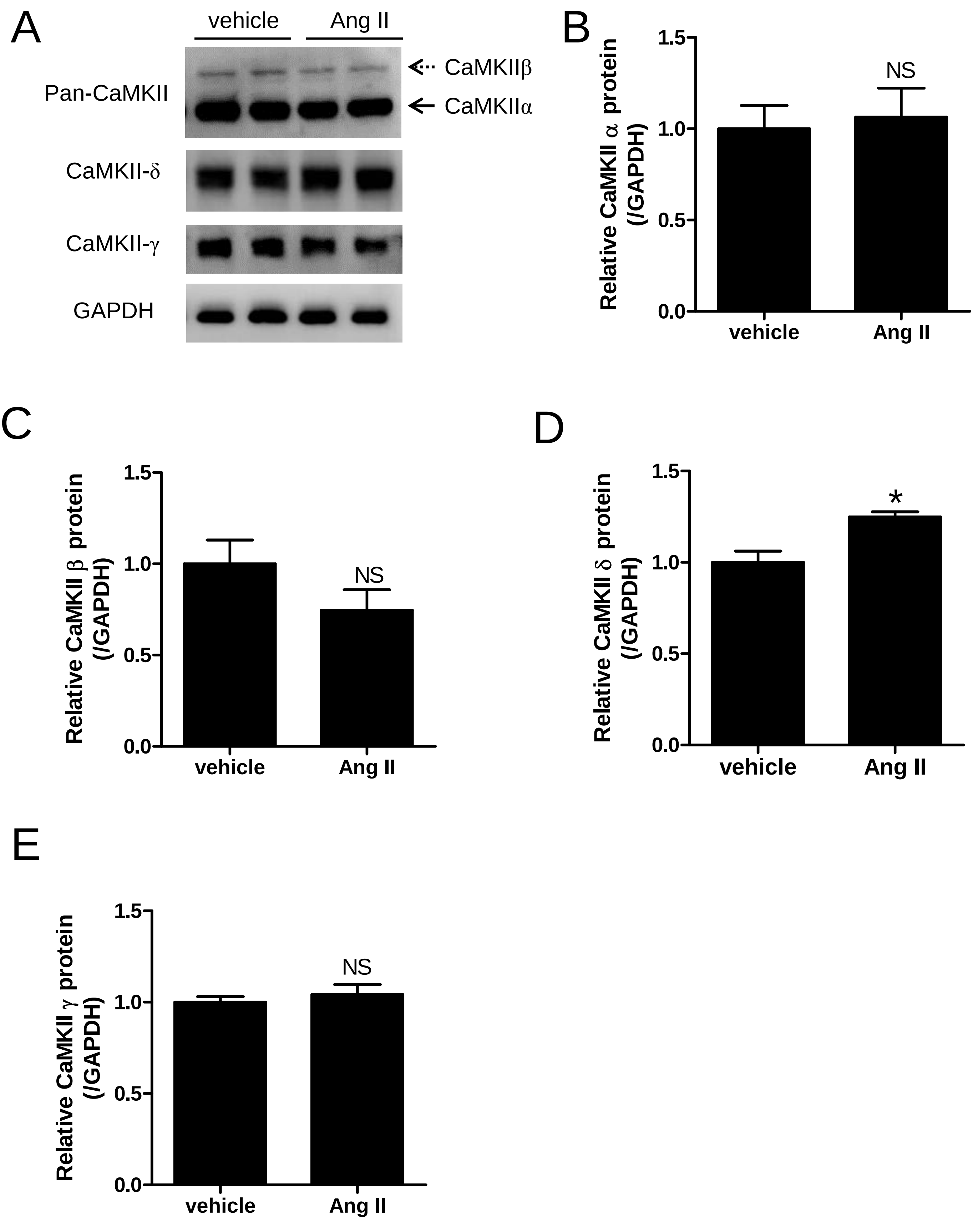

Supplementary Figure 3

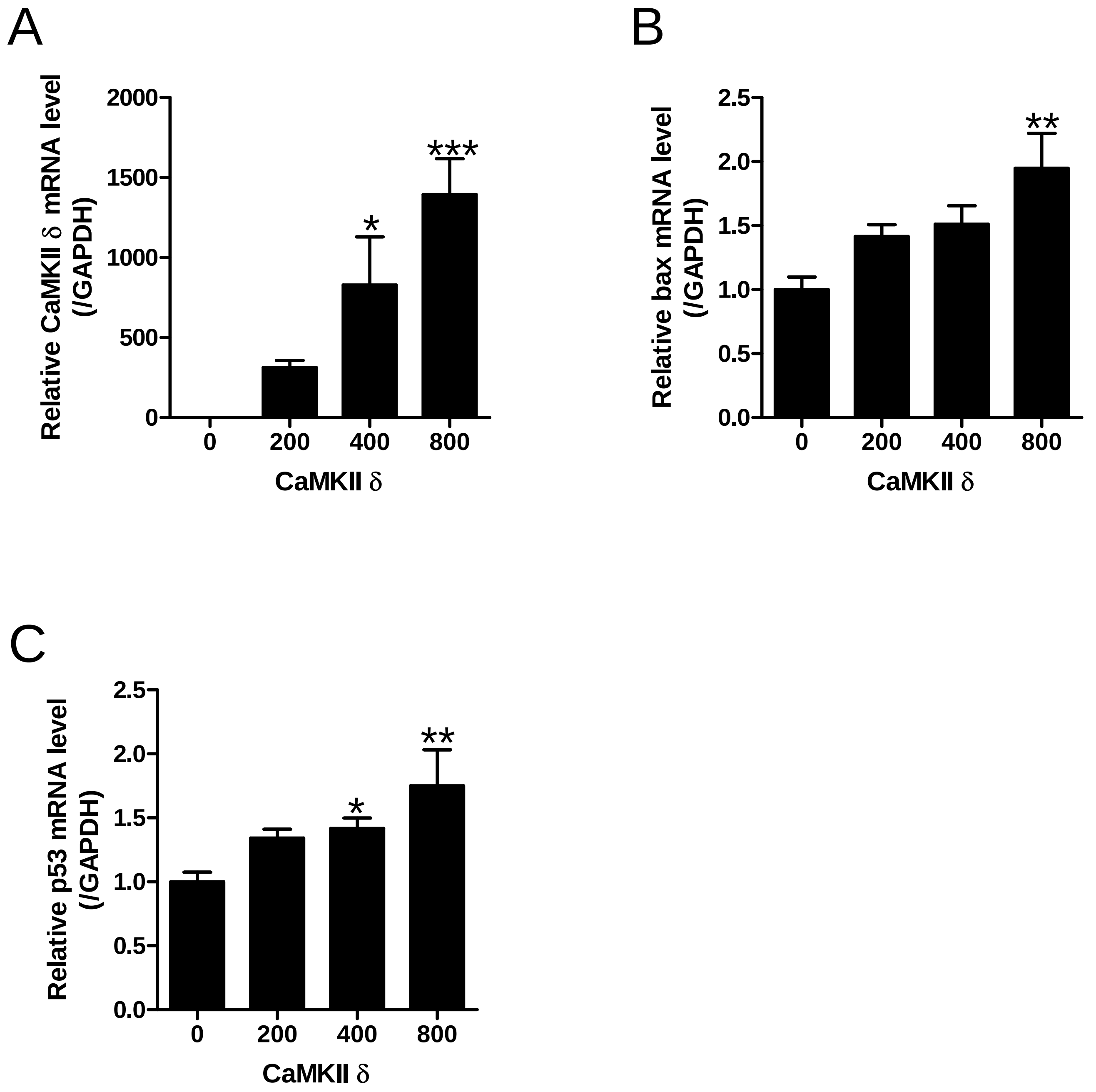

Supplementary Figure 4

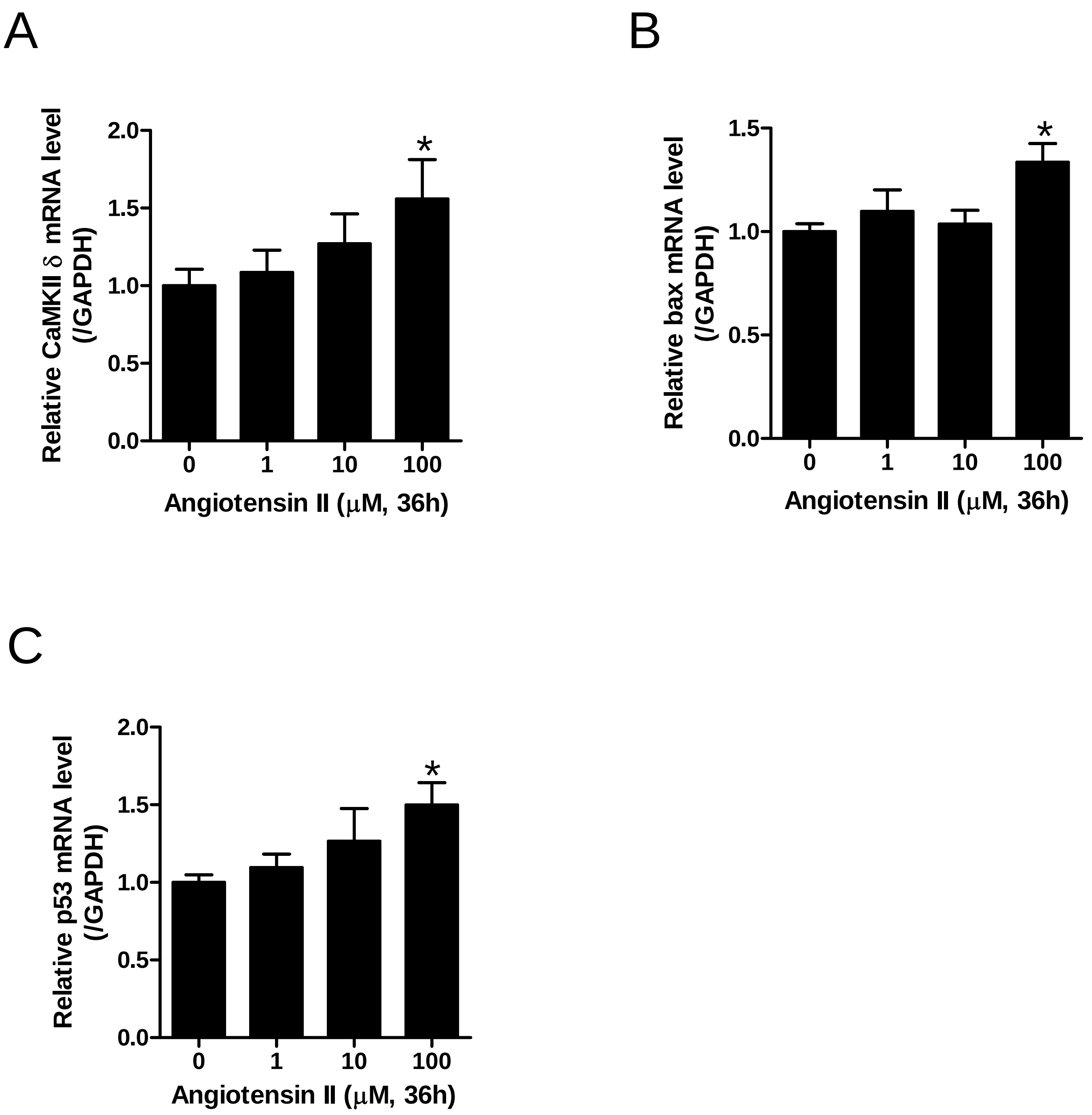

Supplement: Supplementary file 1 — Fig S1. Gallic acid reduces apoptosis in spontaneously hypertensive rats. Fig. S2. CaMKII δ protein levels are increased in angiotensin II‐treated H9c2 cells. Fig. S3. The forced expression of CaMKII δ increases CaMKII δ, bax, and p53 mRNA levels in H9c2 cells. Fig. S4. Angiotensin II stimulus increases mRNA levels of CaMKII δ, bax, and p53 in H9c2 cells. [file JCMM-22-1517-s001.pdf]
